# Does the sex ratio of singleton births after frozen single blastocyst transfer differ in relation to blastocyst development?

**DOI:** 10.1186/s12958-020-00623-x

**Published:** 2020-07-15

**Authors:** Hua Lou, Na Li, Xiaoke Zhang, Ling Sun, Xingling Wang, Dayong Hao, Shihong Cui

**Affiliations:** 1 Reproductive Center, the Third Affiliated Hospital of Zhengzhou University, Zhengzhou, 450052 Henan Province China; 2 Department of Obstetrics and Gynecology, the Third Affiliated Hospital of Zhengzhou University, Zhengzhou, 450052 Henan Province China

**Keywords:** Blastocyst, Frozen-thawed transfer, Inner cell mass, Trophectoderm, Sex ratio

## Abstract

**Purpose:**

To investigate the associations between blastocyst development and the sex ratio (male:female) among singleton live births resulting from single-blastocyst frozen embryo transfer (FET) cycles.

**Methods:**

Patients with singleton live births following the first autologous single FET of non- preimplantation genetic testing (PGT) blastocysts in a single reproductive medicine department between January 2015 and February 2019 were included in this retrospective study. The primary outcome measure was the singleton sex ratio. Multivariable logistic regression models were used to estimate the associations between blastocyst quality and singleton sex ratio after adjustment for some potential confounders.

**Results:**

There were 638 high-quality and 572 poor-quality single blastocyst FETs, and the blastocysts were conceived via 855 IVF and 355 ICSI treatments. A total of 1210 singleton live births were assessed. High-quality single blastocyst FET resulted in a significantly higher sex ratio than did poor-quality single blastocyst FET (60% vs. 49.7%, *P* < 0.001). The infertility cause was not associated with sex ratio among singleton live births (*P* = 0.537). The results of a multivariate analysis revealed that a high-quality blastocyst has a 150% higher probability of being male than a poor-quality blastocyst (adjusted odds ratio (aOR) 1.57; 95% CI 1.24–2, *P <* 0.001). Among the three blastocyst morphological parameters, Grade B trophectoderm was significantly associated with a higher sex ratio than Grade C (aOR 1.71; 95% CI 1.33–2.21. *P* < 0.001). Neither expansion degree nor inner cell mass degree were significantly associated with the singleton sex ratio.

**Conclusions:**

A single high-quality blastocyst FET has a higher chance of resulting in a male infant than a female infant. The results demonstrate that grade B trophectoderm confers benefits in improving the implantation potential of male blastocysts.

## Introduction

Ideally, human-assisted reproductive technology (ART) aims to achieve a single live birth. Following improvements in blastocyst culture systems and freezing technology [1, 2], single blastocyst transfer (SBT) is now considered the most effective means of avoiding multiple pregnancies subsequent to ART [3–6]. Gradually, SBT has become the preferred transfer strategy throughout the world [7–10]. However, blastocyst transfer has some potential limitations, including adverse effects such as a male-biased imbalance in the sex ratio and an increased incidence of monozygotic twinning (MZT) [11–13].

The sex ratio at birth is often calculated as the proportion of males among all live births or the number of male births per 100 female births [14]. Dean et al. reported a significantly higher sex ratio at birth among infants born after SBT (54.1%) than among those born after cleavage-stage transfer cycles (49.9%) [15]. Another study determined that the higher sex ratio was independent of the fertilization method [16]. However, the details of the relationship between the characteristics of blastocysts and the sex ratio at birth remain unclear.

Blastocyst grading prior to embryo transfer usually occurs on day 5 or day 6 of culture. A detailed blastocyst scoring system is required to identify the blastocysts with the highest implantation potential. Static morphology remains the most commonly used embryo selection method worldwide. This system is used to grade blastocysts according to three different variables: blastocoel expansion, inner cell mass and trophectoderm [17]. Several studies have attempted to identify the individual contributions of these parameters to the implantation potential or live birth rate. In particular, trophectoderm morphology may be predictive of pregnancy outcomes related to single blastocyst transfer [18].

Ebner et al. reported that the embryo sex ratio was skewed significantly in relation to morphology. Male blastocysts had a 2.53-fold higher odds of receiving a trophectoderm quality score of A than female blastocysts [19]. All previous related studies were based on morphological parameters and embryo sex. To our knowledge, no previous study has explored the relationship between blastocyst development and the sex ratio of singleton live births after single-blastocyst frozen embryo transfer (FET) cycles. The objective of this study was to investigate this association and fill this gap in the literature.

## Materials and methods

### Study subjects

**Fig. 1:**
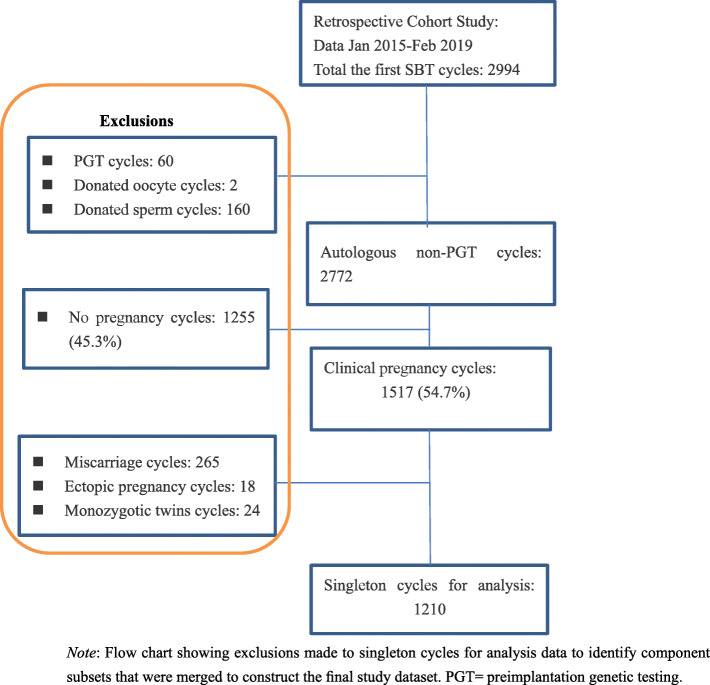
Data selection process for analysis of perinatal outcomes in singleton live births as a result of the first single blastocyst frozen embryo transfer (FET) cycles. *Note*: Flow chart showing exclusions made to singleton cycles for analysis data to identify component subsets that were merged to construct the final study dataset. PGT = preimplantation genetic testing

Patients with singleton live births following the first autologous single FET of non- preimplantation genetic testing (PGT) blastocysts at the Third Affiliated Hospital of Zhengzhou University between January 2015 and February 2019 were included in this retrospective study. Only the first FET cycle of each patient was analyzed to avoid repeated measures bias. Patients who underwent PGT, used donor oocytes or donor sperm were excluded. The study sample identification and collation process is illustrated in Fig. 1. The data collection protocol for this study was approved by our Institutional Review Board.

### Ovarian stimulation protocol

Each female patient underwent a conventional ovarian hyperstimulation procedure involving a gonadotrophin-releasing hormone agonist or antagonist. The physician adjusted the starting dose according to the patient’s age, body mass index (BMI) and ovarian reserve. Ovarian follicle development was monitored based on serum estradiol and transvaginal ultrasonographic measurements. Oocytes were retrieved transvaginally 36–38 h after human chorionic gonadotrophin (Serono, Aubonne, Switzerland) administration when at least 40% of the follicles had reached or exceeded an average diameter of 18 mm as determined by ultrasound. The follicles were aspirated using a single-lumen needle attached to a syringe under transvaginal ultrasound guidance. The oocytes were then inseminated via conventional in vitro fertilization (IVF) or intracytoplasmic sperm injection (ICSI).

### Laboratory protocol

Embryos were placed into the incubator (K-MINC-1000, Cook, United States) and cultured at 6% CO_2_, 5% O_2_ and 37 °C. G-1™ plus (Vitrolife, Sweden) was used to culture the embryos from the pronucleate stage to day 3. Morphological evaluation of the cleavage-stage embryos was performed on day 2 and day 3 based on the number of blastomeres, rate of fragmentation, multinucleation of the blastomeres and early compaction [20]. The two best-quality cleavage embryos were chosen for fresh cycle transfer or cryopreservation on day 3, with surplus embryos being cultured to the blastocyst stage for possible cryopreservation.

Blastocyst morphology was evaluated before cryopreservation according to the classification devised by Gardner and Schoolcraft [21] on day 5 or day 6 after insemination. As a general rule, the inner cell mass quality should be at least B to optimize cryosurvival. High-quality blastocysts were defined as those with a blastocoel grade > B3, inner cell mass grade A/B and trophectoderm grade A/B (AA, AB, BA, BB). Poor-quality blastocysts were defined as those with a blastocoel grade > B3, inner cell mass grade A/B and trophectoderm grade C (AC, BC) [22].

Before vitrification, fully expanded blastocysts were collapsed artificially using a laser [23, 24]. After collapsing the blastocoel, the shrunken blastocyst was vitrified using a Cryotop device (Kitazato BioPharma Co. Shizuoka, Japan). The media employed were vitrification and warming kits. For vitrification, the blastocysts were first equilibrated in solution I [7.5% v/v ethylene glycol (EG) and 7.5% v/v dimethyl sulfoxide (DMSO)] at room temperature for 10 min and then placed into vitrification solution II (15% v/v EG, 15% v/v DMSO and 0.5 M sucrose) for 1 min. Subsequently, blastocysts were individually loaded onto Cryotops in a volume of < 0.1 μl and quickly plunged into liquid nitrogen. Warming was performed by placing the Cryotop in the thawing solution (1.0 M sucrose) for 1 min at 37 °C. Then, the blastocyst was moved to the dilution solution (0.5 M sucrose) for 3 min at room temperature, followed by two steps in washing solution at room temperature for 5 min each. The blastocyte was then transferred into a 50-μL droplet of culture medium (G_2_; Vitrolife) under mineral oil. Blastocysts were transferred 1–2 h postwarming.

### Embryo transfer and clinical outcomes

FET was performed after preparation via hormone replacement therapy (HRT) or during a natural cycle. Vaginal or oral progesterone (Crinone, Merck Serono, Switzerland) was provided for luteal support. On day 6 of progesterone administration, a single vitrified blastocyst was selected for transfer based on morphology grading. All transfer procedures were directed by ultrasound guidance as previously described [25]. Only single blastocysts were selected for transfer. No blastocyst transfer was performed if the endometrial thickness was < 7 mm. A clinical pregnancy was confirmed by the ultrasonographic visualization of an intrauterine gestational sac with fetal heart activity at 4 weeks after blastocyst transfer. In this study, the primary outcome measure was the singleton sex ratio. The sex ratio at birth was calculated as the proportion of males among all live births.

### Statistical analysis

All data analyses were performed using the SPSS 25.0 statistical software package. Continuous data are presented as the mean and standard deviation (SD). Differences between two categorical variables were analyzed using the chi-square test depending on the data distribution. A multivariate logistic regression analysis with adjustment for major covariates (age, body mass index, type of fertilization and day of embryo transfer) was used to assess whether the sex ratio of singleton live births was affected by various morphological parameters used for grading. The data are reported as adjusted odds ratios (ORs) and 95% confidence intervals (95% CIs). A *P* value < 0.05 was set as the threshold of statistical significance.

## Results

### Demographics and basic characteristics

The final study dataset consisted of *n* = 1210 validated singleton live births. An analysis of the collected demographic and clinical data revealed that there were 638 (52.7%) high-quality and 572(47.3%) poor-quality single blastocyst FETs, and blastocysts were conceived via 855 (70.7%) IVF and 355 (29.3%) ICSI treatments. The analyzed cycles yielded 667 male infants and 543 female infants. The sex ratio was 55.1% (667/1210). The baseline characteristics of the included treatment cycles are described in Table 1.

**Table 1:** The characteristics of 1210 patients included the sex ratio among singleton births following the first single blastocyst frozen embryo transfer (FET) cycles performed from January 2015 to February 2019

Characteristic	Description
Number of cycles	1210
Mean maternal age (years±SD)	30.5 ± 4.5
Mean paternal age (years±SD)	31.6 ± 5.4
Mean maternal BMI (kg/m^2^)	23.6 ± 3.2
Mean duration of infertility (years±SD)	3.3 ± 2.6
Type of infertility (%)	
Primary	541 (44.7%)
Secondary	669 (55.3%)
Main infertility cause (%)	
Female factor	661 (54.6%)
Male factor	307 (25.4%)
Mixed factor	193 (16%)
Unexplained infertility*	49 (4%)
Type of fertilization (%)	
IVF	855 (70.7%)
ICSI	355 (29.3%)
Day of embryo transfer (%)	
D5	783 (64.7%)
D6	427 (35.3%)
Blastocyst quality of transfer (%)	
Good	638 (52.7%)
Poor	572 (47.3%)
Singleton livebirths (%)	
Male	667 (55.1%)
Female	543 (44.9%)

*Note*: Data are presented as the mean ± SD and proportion (%). BMI = body mass index; D = day; IVF = in vitro fertilization; ICSI = intracytoplasmic sperm injection; *Unexplained infertility is rarely used as a diagnostic indicator for IVF in China because it is a controversial diagnosis

**Fig. 2:**
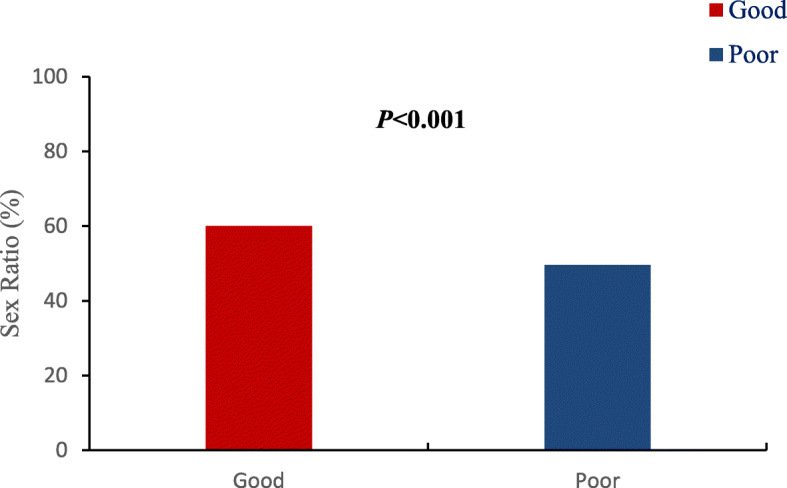
Sex ratio among singleton births following single blastocyst frozen embryo transfer (FET) cycles with different blastocyst qualities

In the present study we checked the possible influence of patient characteristics and the embryo culture condition on the sex ratio. We found that high-quality single blastocyst FET resulted in a significantly higher sex ratio than did poor-quality single blastocyst FET (60% vs. 49.7%, *P* < 0.001, Fig. 2.). Maternal age, maternal BMI, main infertility cause, type of fertilization or day of embryo transfer had no significant influence on the sex ratio as listed in Table 2.

**Table 2:** Sex ratio among singleton live births stratified by different demographic characteristics and the embryo culture condition

Characteristic	Singletons (n)	Male (n)	Sex ratio(%)	*P*-value
Blastocyst quality				<0.001
High	638	383	60.0	
Poor	572	284	49.7	
Maternal age				0.261
<35	971	543	55.9	
≥ 35	239	124	51.9	
Maternal BMI				0.173
<18.5	47	32	68.1	
18.5 ~ 24.9	772	425	55.1	
≥ 25	391	210	53.7	
Type of infertility				0.887
Primary	541	297	54.9	
Secondary	669	370	55.3	
Cause of infertility				0.537
Female factor	661	363	54.9	
Male factor	307	178	58	
Combined factor	193	102	52.8	
Unexplained	49	24	49	
Type of fertilization				0.191
IVF	855	461	53.9	
ICSI	355	206	58.0	
Day of frozen embryo				0.774
D5	783	434	55.4	
D6	427	233	54.6	

*Note*: The sex ratio at birth is calculated as the proportion of males among all livebirths. *P* < 0.05 was considered statistically significant. CI = confidence interval; BMI = body mass index; D = day; IVF = in vitro fertilization; ICSI = intracytoplasmic sperm injection

After adjustment for the potential confounder factors, we identified significant associations of the sex ratio among singleton births with the morphological characteristics of blastocysts. The results of a multivariate analysis revealed that a high-quality blastocyst has a 150% higher probability of being male than a poor-quality blastocyst (aOR1.57; 95% CI 1.24–2, *P <* 0.001). Particularly, when the trophectoderm was of Grade B, the blastocysts showed a 170% higher probability of being male than when the trophectoderm was of Grade C (aOR1.71; 95% CI 1.33–2.21, *P <* 0.001). Neither expansion degree or inner cell mass degree were not significantly associated with the singleton sex ratio (Table 3).

**Table 3:** Multivariate logistic regression model of sex ratio among singleton births following single blastocyst frozen embryo transfer (FET) cycles in relation to blastocyst quality and morphology

Variable	aOR(95% CI)	*P* value
Good	1.57 (1.24 ~ 2)	< 0.001
Poor	Reference	
Expansion degree		
4	Reference	
5	0.96 (0.61 ~ 1.49)	0.843
6	1.63 (0.89 ~ 2.99)	0.111
Inner cell mass		
A	0.71 (0.49 ~ 1.01)	0.059
B	Reference	
Trophectoderm		
A	1.4 (0.91 ~ 2.16)	0.128
B	1.71 (1.33 ~ 2.21)	< 0.001
C	Reference	

*Note*: Sex ratio adjusted for age, BMI, type of fertilization, day of embryo transfer. *P* < 0.05 was considered statistically significant. BMI = body mass index; CI = confidence interval

**Fig. 3:**
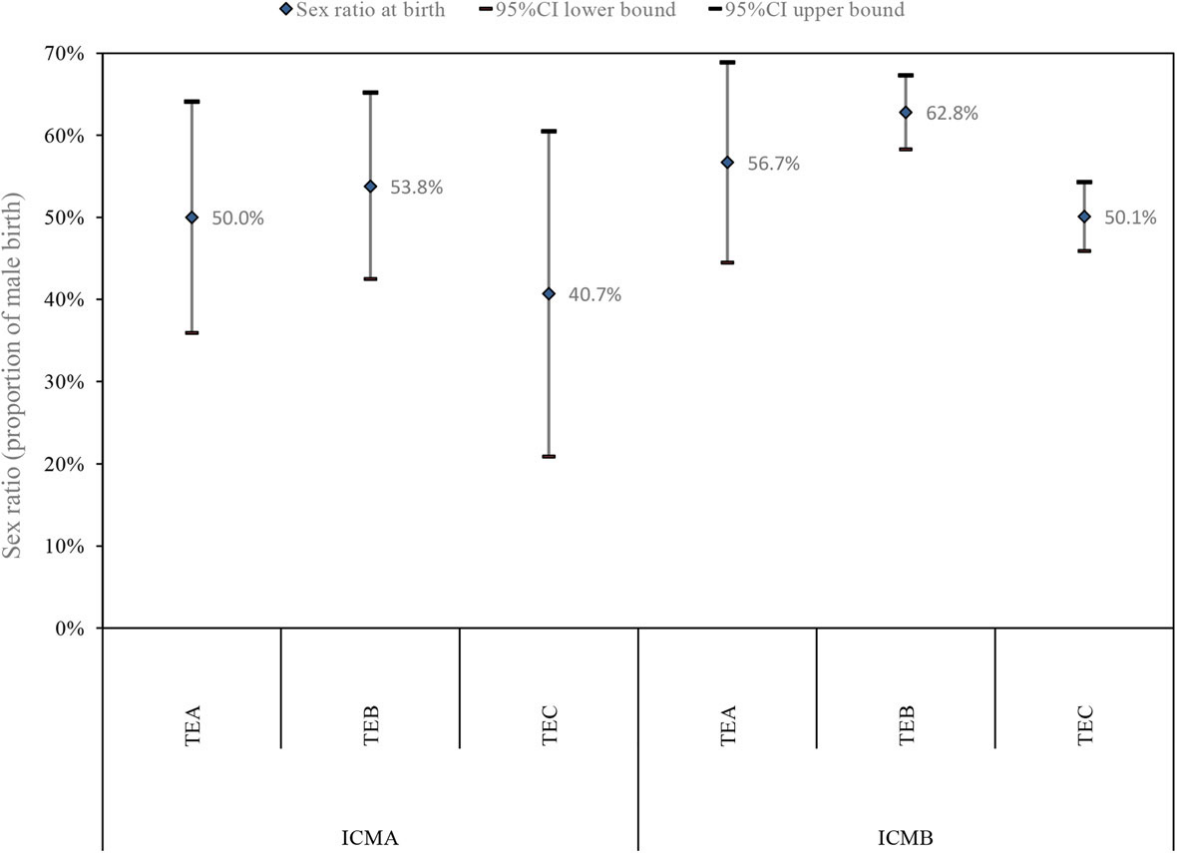
Sex ratio among singleton births following single blastocyst frozen embryo transfer (FET) cycles by different grades of trophectoderm (TE) and inner cell mass (ICM). *Note*: Sex ratio adjusted for age, BMI, type of fertilization, day of embryo transfer. *P* < 0.05 was considered statistically significant. CI = confidence interval; TE = trophectoderm; ICM = inner cell mass

To further assess the effect of the trophectoderm grades on the sex ratio among singleton live births, we stratified the data according to inner cell mass grade, as shown in Fig. 3. In both the inner cell mass Grade A and B groups, Grade B trophectoderm showed the highest sex ratio. In the inner cell mass Grade B group, when the trophectoderm was of Grade B, the blastocysts showed a 175% higher probability of being male than when the trophectoderm was of Grade C (odds ratio1.75; 95% CI1.34–2.29, *P* < 0.001).

## Discussion

Our study results indicate that implantation with high-quality blastocysts, rather than poor-quality blastocysts, during single-blastocyst FET cycles is associated with an increased sex ratio among live births. We further determined that only the trophectoderm grade was associated with a significantly higher sex ratio among singleton live births. Similar observations were not observed for the blastocoel expansion and inner cell mass grades. Therefore, our findings demonstrate that a grade B trophectoderm is associated with a higher probability that a male infant will be born from a single-blastocyst FET cycle.

Single blastocyst transfer was designed to avoid the complications associated with multiple pregnancies and is thus becoming the preferred type of ART. The use of extended blastocyst culture conditions may favor the selection of male blastocysts for transfer, as male embryos are thought to exhibit greater preimplantation developmental rates. In this study, the sex ratio among single-blastocyst FET cycles was 55.1%, which was consistent with the ratios reported in previous studies. For example, a large nationwide longitudinal birth cohort study of 103,099 pregnancies in Japan reported a skewed sex ratio in favor of males after blastocyst transfer relative to spontaneous conception (OR: 1.095; 95% CI: 1.001–1.198). However, the sex ratios in the non-ART treatment and cleavage groups were slightly lower than or equivalent to the ratio in the spontaneous conception group [13]. A meta-analysis of 13 studies also suggested that blastocyst transfer was associated with a male-biased sex ratio when compared with cleavage-stage embryo transfer (OR: 0.89, 95% CI: 0.86–0.93) [26]. This observation may be attributable to the more frequent selection of male embryos for transfer, as male embryos develop more rapidly in vitro and thus may appear more viable at the blastocyst stage [27, 28]. We were interested in determining the mechanism underlying the observed phenomenon of sex deviation in offspring after blastocyst transfer. Accordingly, we retrospectively analyzed the potential effects of morphological parameters on the sex ratio after single-blastocyst FET.

Our study is the first to correlate various blastocyst parameter grades with the sex ratio among singleton live births after single-blastocyst FET cycles. We observed a significant increasing trend in the sex ratio in the context of high-quality versus poor-quality blastocyst transfer, suggesting that the transfer of more advanced blastocysts may increase the proportion of male infants. We note that male blastocysts may grow more rapidly and receive better morphological scores than female blastocysts. These discrepancies may at least partially account for the skewed sex ratio at birth. Mohamad et al. reported a greater likelihood of euploidy among blastocysts with good morphology scores and among embryos that progressed more rapidly to the blastocyst stage [29]. Therefore, differences in the observed sex ratios of live births subsequent to single-blastocyst FET cycles may also be attributable to blastocyst selection and transfer strategies.

We did not find that delayed blastulation had a significant effect on the newborn sex ratio, as similar values were observed for the groups that received day 5 and day 6 embryo transfers. Our results are consistent with those of a study published by El-Toukhy et al., which reported similar live birth rates after the transfer of vitrified embryos on day 5 and day 6 [30]. Desai N et al. used a morphokinetics approach combined with preimplantation genetic screening test models to determine that embryos exhibiting delayed blastulation were more likely to exhibit aneuploidy [31]. Blastocyst morphology, which is based on blastocoel expansion, the trophectoderm and the inner cell mass, must also be considered when evaluating the outcomes of FET. Initially, we observed an association between the degree of blastocoel expansion and the sex ratio among singleton live births in a small sample. However, we did not observe any association between these variables. Samer et al. reported that sex-related differences in development are highly significant, such that male embryos were 2.6-times more likely to produce a grade 5 or 6 blastocyst than female embryos [32]. Our study differed from that study because we focused on the sex ratio at birth rather than in the embryos. The functions of blastocoel expansion involve hatching from the zona pellucida, adhesion to and invasion of the endometrium and establishment of maternal communication [32, 33]. In a recent study, Jing Zhao et al. confirmed that the degree of blastocoel expansion was a better predictor than the trophectoderm or inner cell mass grade in terms of the likelihood of live birth after both single-blastocyst fresh transfer and FET cycles [34]. The degree of blastocoel expansion may be an essential factor in a successful pregnancy and should be prioritized when selecting a frozen blastocyst for transfer [35].

Some previous studies have emphasized the importance of the inner cell mass with respect to pregnancy establishment [36, 37]. Irani et al. applied the same embryo-grading criteria used in the analysis to 477 preimplantation-genetic-testing-confirmed single-euploid blastocyst FET cycles. These authors determined that the overall blastocyst quality and inner cell mass grade were the most effective predictors of a successful pregnancy and suggested the use of both parameters to facilitate the selection of high-quality euploid blastocysts for IVF transfer [29]. In contrast, Ahlström et al. emphasized the higher predictive strength of the trophectoderm grade relative to the inner cell mass grade when selecting the optimal blastocyst for both fresh and frozen thawed cycles [38]. In our study, however, we did not find that the inner cell mass quality had a significant effect on the newborn sex ratio after single-blastocyst FET cycles.

In our analysis, the trophectoderm grade was identified as the most important factor affecting the newborn sex ratio after a single-blastocyst FET cycle. A significant association was observed between grade B trophectoderm and sex ratio among infants conceived via the transfer of blastocysts. In other words, the effect of blastocyst morphology on the sex ratio is only obvious when blastocysts with grade B trophectoderm are selected. The importance of the trophectoderm during the critical implantation phase might be ascribed to its role as a promoter of hatching and endometrial invasion [39, 40]. According to Thomas et al., the observed correlations of trophectoderm performance with infant sex and the rates of implantation, pregnancy and pregnancy loss indicate that this measure will eventually be prioritized over the inner cell mass score [19]. Interestingly, we also demonstrated that after controlling for the inner cell mass, trophoblast grade B was positively correlated with the highest sex ratio among singleton births. Disparities in morphological scores between male and female embryos are unlikely to reflect any difference in competence. However, these variations clearly emphasize the tendency of male embryos to reach the final stages of blastocyst development more rapidly than female embryos [31].

This study has several strengths. First, all cycles and cryopreservation procedures were completed at a single institution according to a strict cycle protocol. Embryologists scored several standardized transfer parameters using a consistent system according to the classification devised by Gardner and Schoolcraft [21]. We also included only single-blastocyst FET cycles that resulted in singleton live births to enable us to control the within-transfer characteristics that are known to affect outcomes. Our aim was to isolate morphological blastocyst parameters as the exposure while regulating several other potential confounders to the greatest extent possible.

However, our study also has some limitations. First, this was a retrospective study of a relatively small number of patients treated at a single center. In the future, a study with a large sample size is needed for validation. Second, further analyses of blastocyst grading according to morphological parameters should investigate dynamic parameters by time-lapse microscopy, which allows the assessment of embryo morphodynamic patterns throughout preimplantation development. Third, our strategy required that the two best-quality cleavage embryos be chosen for fresh cycle transfer or cryopreservation on day 3, with surplus embryos being cultured to the blastocyst stage for possible cryopreservation. The result was that the blastocyst used for culture was a suboptimal embryo. Therefore, we had very few good embryos (AA) and a large percentage of fair BB and poor BC embryos, and the strategy required an inner cell mass grade of B or higher for optimal cryosurvival. Therefore, we were only able to evaluate the outcomes associated with an inner cell mass grade of A or B. Fourth, blastocyst transfer is more advantageous than cleavage-stage embryo transfer in terms of successful implantation从 and pregnancy rates. We prefer high-quality blastocyst transfer to obtain a better clinical pregnancy rate. Although we determined that the transfer of high-quality blastocysts may skew the sex ratio in favor of male embryos, it is difficult to change perceptions regarding popular blastocyst transfer strategies.

## Conclusion

In summary, we demonstrated that the morphological criteria associated with higher-quality blastocysts result in a higher sex ratio after single-blastocyst FET. However, we found that only the trophectoderm grade was significantly associated with the sex ratio among offspring. Our findings may elucidate the underlying determinants of the skewed sex ratio after blastocyst transfer. Clinicians should be aware of the effects of certain protocols on sex distribution, given recent trends towards the increased use of blastocyst transfer. In the future, we aim to identify additional markers of embryonic implantation potential, with the aim of maintaining the equilibrium of the sex ratio among offspring without affecting the pregnancy rate.

## Data Availability

The datasets used and/or analyzed during the current study are available from the corresponding author on reasonable request.

## Abbreviations

ART: Assisted reproductive technology
FET: Frozen embryo transfer
BMI: Body mass index
IVF: In vitro fertilization
ICSI: Intracytoplasmic sperm injection
HRT: Hormone replacement therapy
OR: Odds ratio
95% CI: 95% confidence interval
ICM: Inner cell mass
TE: Trophectoderm
